# Sentinel lymph node mapping in endometrial cancer: A comparison of main national and international guidelines

**DOI:** 10.1002/ijgo.14307

**Published:** 2022-07-05

**Authors:** Aharon Dick, Tamar Perri, Liron Kogan, Benny Brandt, Raanan Meyer, Gabriel Levin

**Affiliations:** ^1^ The Department of Obstetrics and Gynecology, Hadassah Medical Center, Faculty of Medicine Hebrew University of Jerusalem Jerusalem Israel; ^2^ The Department of Gynecologic Oncology, Hadassah Medical Center, Faculty of Medicine Hebrew University of Jerusalem Jerusalem Israel; ^3^ Department of Obstetrics and Gynecology Chaim Sheba Medical Center Ramat‐Gan Israel; ^4^ Faculty of Medicine, Tel‐Aviv University Tel‐Aviv Israel

**Keywords:** endometrial cancer, indocyanine green, lymphadenectomy, mapping, sentinel lymph node, Ultrastaging

## Abstract

**Objectives:**

To compare national and international guidelines regarding sentinel lymph node (SLN) mapping in endometrial cancer.

**Methods:**

A descriptive comparative study of the National Comprehensive Cancer Network (NCCN), the Society of Gynecologic Oncology (SGO), the European Society of Gynecological Oncology (ESGO), the British Gynecological Cancer Society (BGCS), and the Japan Society of Gynecologic Oncology (JSGO) guidelines.

**Results:**

There is a broad consensus that SLN mapping is an appropriate alternative to pelvic lymphadenectomy for uterine‐confined endometrioid endometrial cancer (five of five guidelines). It is broadly accepted that a full lymphadenectomy should be performed in case of failed SLN mapping (four of five guidelines), and that mapping with the fluorescent dye indocyanine green is superior to other methods (four of five guidelines). It is agreed that the cervix is the preferable site for dye injection (four of five guidelines), and pathology ultrastaging is advocated by most guidelines (three of five guidelines). Regarding high‐risk patients (i.e., high‐grade histology and non‐endometroid carcinomas), some guidelines accept (three of five), but others currently do not advocate (one of five guidelines), SLN mapping as a sole method for lymph node evaluation. There is no consensus regarding para‐aortic lymph node evaluation in pelvic SLN‐positive patients.

**Conclusion:**

Guidelines for SLN mapping are comparable with regards to surgical technique, ultrastaging, and management in case of failed mapping. Nevertheless, some variations exist regarding the management of high‐grade histology and positive pelvic lymph nodes.

## Synopsis

Main national and international guidelines on sentinel lymph nodes in endometrial cancer are mostly similar, with some variations regarding high‐grade histology and positive pelvic lymph nodes.

## 1 INTRODUCTION

Endometrial cancer is the most common gynecologic malignancy in high‐income countries, with an estimated incidence of 66 570 newly diagnosed cases in 2021 in the USA, leading to 12 940 deaths annually. Globally, 382 069 new cases of endometrial cancer were diagnosed in 2018, with 89 909 deaths worldwide.^1^, ^2^

Surgery is an essential component in the treatment of endometrial cancer. Hysterectomy, usually with bilateral salpingo‐oophorectomy, is the mainstay of management. However, the approach to lymph node staging remains controversial.

In early‐stage endometrial cancer, systematic pelvic and para‐aortic lymphadenectomy does not affect oncologic outcomes, including overall survival and disease‐free survival, but increases perioperative morbidity, operative time, and costs.^3^, ^4^ Over the past few years, less‐invasive strategies for lymph node evaluation have been investigated. Sentinel lymph node (SLN) sampling, first described in 1996, was established to be as accurate as systematic lymphadenectomy in evaluating the nodal status of early endometrial cancer and is now the standard of care in most gynecologic oncology units.^5^, ^6^

With the global and rapid adoption of SLN sampling, a wide variability of surgical techniques and local protocols were suggested.^5^ International societies developed guidelines to minimize variations in practice and to improve outcomes.^6^

The purpose of this study was to summarize and compare the points of consensus and controversy of guidelines for SLN mapping in endometrial cancer in leading national and international societies.

## 2 MATERIALS AND METHODS

This was a descriptive comparative study. We accessed the websites of the world's major gynecologic oncology societies and retrieved any publications on SLN mapping in the management of endometrial cancer. The following guidelines were searched: the National Comprehensive Cancer Network (NCCN),^7^ the Society of Gynecologic Oncology (SGO),^6^ the European Society of Gynecological Oncology (ESGO),^8^ the British Gynecological Cancer Society (BGCS),^9^ and the Japan Society of Gynecologic Oncology (JSGO).^10^

### 2.1 ETHICS STATEMENT

Institutional review board approval to conduct this study was not required as only previously published documents were analyzed.

## 3 RESULTS

A comparison summary of the characteristics and recommendations of these five guidelines is presented in Table 1.

**TABLE 1:** Summary of results

Parameter	NCCN	SGO	ESGO	BGCS	JSGO
Indications					
Stages I–II (uterine confined) (low/intermediate risk)	May be considered	Can be performed	Can be considered	Can be considered	An option. Omission of full LAD is suggested
High grade (grade 3, clear cell/serous/carcinosarcoma) (intermediate–high/high risk)	Potential alternative to full LAD	Feasible with completion of full LAD + para‐aortic assessment	Acceptable alternative to full LAD in stages I–II	Can be considered	Not mentioned
Techniques—dyes					
Colorimetric mapping	Not mentioned	Least complex. Isofulfan blue: risk for anaphylaxis Methylene blue: risk for methemoglobinemia and serotonin syndrome	Not mentioned	Not mentioned	Not mentioned
Radionuclear method: Tc99	Most commonly used	When combined with colorimetric‐ optimal detection. Advantageous in women with fatty nodal basin + cases of unpredictable lymphatic drainage	Not mentioned	When combined with colorimetric‐ highest detection rate (equal to ICG)	Not mentioned
Near infrared method: ICG dye	Very high detection rate	Preferable because of technical ease+ high success + reliability. Risk for anaphylaxis	Highest detection rate	Highest detection rate (equal to Tc99 + colorimetric)	Not mentioned
Injection site	Cervix—superficial and deep	Cervix—most favored. Location: submucosa or superficial	Cervix	Cervix	Not mentioned
Failed mapping	Side‐specific LAD	Side‐specific LAD	Side‐specific LAD in intermediate–high−/high‐risk patients	Full LAD (when lymph node staging is indicated)	Not mentioned
Ultrastaging	Important for detection of low volume metastasis	Increases the detection of ITCs and micrometastasis	Is recommended	Should be used	Not mentioned
Positive pelvic SLN	Para‐aortic LND (at attending discretion)	Para‐aortic LND (at attending discretion)	Para‐aortic staging can be considered (imaging or surgery)	Not mentioned	Not mentioned
Frozen section of SLN	Only if suspicious	Only if suspicious	Not mentioned	Not mentioned	Not mentioned
No. references	32	97	72	‐	8

Abbreviations: BGCS, British Gynecological Cancer Society; ESGO, European Society of Gynecological Oncology; ICG, indocyanine green; ITC, isolated tumor cells; JSGO, Japan Society of Gynecologic Oncology; LAD, lymphadenectomy; LND, lymph node dissection; NCCN, National Comprehensive Cancer Network; SGO, Society of Gynecologic Oncology; SLN, sentinel lymph node; Tc99, technetium 99.

### 3.1 SLN indications in endometrial cancer

According to the NCCN guidelines, SLN mapping may be considered for patients with apparent uterine‐confined disease^7^ and may be most appropriate for those at low–intermediate risk for metastasis and those unable to tolerate full lymphadenectomy. Among patients with high‐risk histology (grade 3, serous, clear cell, carcinosarcoma) SLN mapping has been referred to as a potential alternative to full lymphadenectomy. The SGO suggests that SLN sampling can be performed in patients with apparent uterine‐confined grade 1 and 2 ('low‐grade') endometrioid cancers.^6^ However, among high‐grade tumors (grade 3 endometrioid, serous, clear cell, or carcinosarcoma) SLN mapping with a close adherence to the SLN algorithm and an add‐on completion of lymphadenectomy with para‐aortic assessment is ‘an acceptable approach’, until more safety data become available. The ESGO states that SLN mapping can be considered in patients with low−/intermediate‐risk disease and can be omitted in cases without myometrial invasion (level 2A).^8^ In high–intermediate−/high‐risk disease, a full lymph node dissection should be performed, yet SLN mapping is an acceptable alternative to systematic lymphadenectomy for lymph node staging in high–intermediate−/high‐risk disease stages I/II (level 3B). The BGCS^9^ recommends that when lymph node dissection is indicated, if imaging suggests that there is no metastasis, and no obvious extrauterine disease is inspected at surgery, the use of SLN algorithms can be considered, even in high‐risk pathologic types (clear cell, papillary serous, and carcinosarcoma). The JSGO recommends, in general, that for some apparently early‐stage low‐grade (I–II) endometrial cancer patients, SLN mapping is suggested. A full lymphadenectomy is indicated for intermediate‐risk or high‐risk patients. In the presence of negative SLNs, omission of further lymphadenectomy might be considered only in the context of clinical trials.^10^ It is emphasized by all societies that regardless of sentinel mapping, any suspicious lymph node should be removed.

### 3.2 SLN mapping techniques in endometrial cancer

#### Injected substance

The NCCN guidelines mention colored dyes; isosulfan blue, methylene blue, and patent blue sodium, as well as indocyanine green (ICG) and technetium‐99 (Tc99).^7^ The SGO guidelines describe the different methods for lymphatic mapping.^6^ Blue dye injections include the use of 3–5 ml of a 1% solution of isofulfan blue, with anaphylaxis risk of 1%, or methylene blue injection of 2–4 ml of a 1% solution with risk of paradoxical methemoglobinemia and serotonin syndrome. Mapping can be performed 10–20 min after injection. The radionuclear methods, using radiolabeled colloid 1 ml of 1 mCi Tc99, is also described by the SGO, acknowledging that it is often used in synergy with colorimetric methods to optimize the detection rate. Tc99 can be advantageous in women with fatty nodal basin and in cases of unpredictable lymphatic drainage.

The near‐infrared method, using ICG, is also described by the SGO: dilution to 0.5–1.25 mg/ml in sterile water and injection of 2–4 ml. It is stated that ICG is superior to blue dyes, particularly in obese women. The risk of anaphylaxis and contraindications are discussed.

The ESGO considers near‐infrared fluorescence as a method with a higher detection rate in comparison with other (unmentioned) techniques (level 2A)^8^ and mentions methylene blue as inferior to ICG. The BGCS advocates either ICG or a combination of blue dye and Tc99m‐labeled colloid.^9^

#### Injection site

The NCCN guidelines provide figures presenting three different approaches of cervical injection sites according to the different origin of lymphatic channels of the cervix (superficial subserosal, intermediate stromal, deep submucosal). The options are: (1) 3 and 9 o'clock; (2) 2, 5, 7, and 10 o'clock, and (3) 12, 3, 6, and 9 o'clock. Moreover, they provide common and less common locations of SLN .^7^ It is further described that superficial (1–3 mm) and optional deep (1–2 cm) injections are performed. The SGO provides tables describing studies of different injection sites, including subserosal uterine fundus, deeper myometrium, and hysteroscopically guided subendometrial tumor injections, stating that cervical injection has become the most favored location. It is specified that injection should be performed slowly, into the submucosa or superficial cervical tissue.^6^ The ESGO mentions that, in general, the cervix is the preferred injection site,^8^ as does the BGCS.^9^

### 3.3 Failed SLN mapping

SLNs are not identified at surgery in up to 6% of cases.^11^ Several known reasons for mapping failure include lymphatic obstruction by bulky tumor, obesity, and use of blue dye only.^6^ The NCCN working algorithm, also cited by the SGO, recommends side‐specific lymphadenectomy in case of mapping failure.^7^ Intraoperative pathologic assessment of the primary tumor specimen may be used to determine the need for additional lymphadenectomy. The ESGO and BGCS also recommended side‐specific (hemi‐pelvic) systematic lymphadenectomy in high–intermediate−/high‐risk patients if the SLN is not detected on either pelvic side.^12^, ^14^

### 3.4 Ultrastaging

Dissected SLNs are sent to serial sectioning and a review of multiple hematoxylin and eosin (H&E)‐stained slides by the pathologists. No formal evidence‐based guidelines for the pathologic assessment of SLNs in endometrial cancer have so far been published, with regards to the number and intervals between sections, depth of sectioning, and immunohistochemistry use. According to the NCCN guidelines, the two main protocols for ultrastaging are either serial H&E sectioning or immunohistochemical staining, with no advantage to either protocol. According to the NCCN guidelines, these enhanced protocols, combined with SLN mapping, has been shown to increase the detection of nodal metastasis, which may alter the stage and tailor adjuvant therapy recommendations (level 2A).^7^ SGO guidelines quote the algorithm proposed by the group at Memorial Sloan Kettering^11^: an initial evaluation by routine H&E and, if negative, two cuts (one H&E and one cytokeratin AE1/AE3) are inspected.^6^ The SGO further describes three other protocols presented in studies with different section intervals and one protocol involving creating a cytologic smear out of bisected node. According to the SGO, ultrastaging can be dismissed in endometrioid endometrial cancer with no myometrial invasion.^6^ The ESGO recommends preforming SLN ultrastaging for the detection of small metastases that could be missed by standard evaluation, but no specific protocol is mentioned. The BGCS also does not refer to a specific protocol, but rather states that ultrastaging protocols should be utilized.^9^

### 3.5 Frozen section

The NCCN guidelines (and also quoted by the SGO guidelines) state that any suspicious lymph nodes should be sent for frozen sections and the results will affect the decision whether to perform para‐aortic lymph node dissection. However, routine SLN frozen sections are not recommended due to low sensitivity for the detection of metastasis and potential alternation of ultrastaging pathology.

The ESGO, BGCS, and JSGO guidelines do not discuss this topic.

### 3.6 Positive SLNs

The NCCN algorithm for surgical staging of endometrial cancer suggests that para‐aortic lymph node dissection should be performed at the attending's discretion.^7^ Likewise, the SGO guidelines state that completion of para‐aortic dissection should be at the attending surgeon's discretion based on individualized patient characteristics and tumor‐based risk criteria (depth of invasion, histology, and pelvic node status). When para‐aortic dissection is omitted, postoperative imaging should be carried out to evaluate the presence of residual disease.^6^ The ESGO states that para‐aortic staging can be considered, either by imaging or by surgery. They mention that lymph node staging does not have a therapeutic value, but is an indication of the extent of disease and provides information for adjuvant treatment planning.^8^

### References

3.7

The guidelines with the largest number of references are the SGO guidelines (97), followed by the ESGO guidelines (72), and the NCCN guidelines (32). The JSGO guidelines cite the least number of references (eight), whereas the BGCS guidelines cite none. Table 2 categorizes the year of publication of references to less than or equal to 5, 5–10, 10–20, and more than 20 years ago. 95.9% of the references mentioned in the ESGO guidelines date to the last decade, whereas 50% of the JSGO references had been published more than 10 years ago.

**TABLE 2:** References among the various guidelines

	NCCN	SGO	ESGO	BGCS	JSGO
<5 years	31.8%	4.1%	69.4%	No information—did not provide list of references	25%
5–10 years	50%	49.4%	26.3%		25%
10–20 years	18.1%	34%	4.1%		50%
>20 years	‐	12.3%	‐		‐

Abbreviations: BGCS, British Gynecological Cancer Society; ESGO, European Society of Gynecological Oncology; JSGO, Japan Society of Gynecologic Oncology; NCCN, National Comprehensive Cancer Network; SGO, Society of Gynecologic Oncology.

### 3.8 Final recommendations level

All NCCN conclusions mentioned in the body of the guidelines were considered level 2A. The SGO guidelines include seven final recommendations, but do not mention the level of evidence. The ESGO guidelines summarize seven recommendations, of which two are level 2, two are level 3, and three are level 4 (Table 3). The BGCS guidelines are summarized in a 12‐point consensus statement and the JSGO guidelines include one final recommendation, grade C1.

**TABLE 3:** Level of recommendations

NCCN	SGO	ESGO	BGCS	JSGO
Level 2A, 100%	Not mentioned	Level 2A, 28.5%	Not mentioned	Grade C1, 100%
		Level 3B, 28.5%		
		Level 4B, 14.2%		
		Level 4C, 28.5%		

*Notes*: NCCN: Category 2A: based upon lower‐level evidence, there is uniform consensus that the intervention is appropriate. ESGO: Level 2: small randomized trials or large randomized trials with a suspicion of bias (lower methodologic quality) or meta‐analyses of such trials or of trials with demonstrated heterogeneity. Level 3: prospective cohort studies. Level 4: retrospective cohort studies or case–control studies. Grade A: strong evidence for efficacy with a substantial clinical benefit, strongly recommended. Grade B: strong or moderate evidence for efficacy but with a limited clinical benefit, generally recommended. Grade C: insufficient evidence for efficacy or benefit does not outweigh the risk or the disadvantages (adverse events, costs, etc.), optional. JSGO: Grade C1: treatment can be considered, or is suggested, but the evidence is insufficient.

Abbreviations: BGCS, British Gynecological Cancer Society; ESGO, European Society of Gynecological Oncology; JSGO, Japan Society of Gynecologic Oncology; NCCN, National Comprehensive Cancer Network; SGO, Society of Gynecologic Oncology.

## 4 DISCUSSION

We compared the leading societies' guidelines regarding SLN mapping in endometrial cancer. It is agreed that the main role of the SLN procedure is the assessment of lymph node tissue for metastases, for the purpose of surgical staging, as there is probably no therapeutic role for lymphadenectomy.^4^ Rather, the assessment of lymphatic tissue provides valuable data regarding the stage of disease and aids tailoring adjuvant therapy.

SLN mapping was first introduced more than 60 years ago in the management of parotid carcinoma^12^ and evolved through the first successful mapping for penile carcinoma 20 years later^13^ to become the standard of care for cutaneous melanoma and breast carcinoma. For endometrial cancer, SLN mapping was first reported more than two decades ago,^14^ using injection of blue dye into the subserosa of the myometrium. SLN mapping is currently considered as an alternative to lymphadenectomy in at least certain surgical staging procedures for endometrial cancer and it is today widely accepted and practiced. However, prospective data on oncologic outcomes, especially in high‐risk endometrial cancer, are as yet lacking. Hence, although one of the objectives of clinical practice guidelines is to improve and standardize the management of the disease, considerable variation may exist among professional societies in both the recommendations and the clinical practices. Despite NCCN guidelines, which are established by an expert panel and are considered the standard for quality cancer care, national guidelines are published to guide local practice in light of available resources, knowledge, and probably legal and litigation issues. Comparing the guidelines of the major societies with regard to SLN mapping in endometrial cancer, it is evident that although most issues are in agreement, some are still controversial.

All guidelines included in the current summary agree that when lymph node assessment is indicated, SLN mapping is advocated for patients with early‐stage endometrial cancer (no disease outside of the uterine corpus). Regarding the role of SLN mapping in the other risk groups of endometrial cancer, the guidelines are in consensus that it is appropriate for low–intermediate‐risk women. Yet, as per SLN in high‐risk endometrial cancer, e.g. high‐grade histology and non‐endometrioid, all guidelines searched less advocate this procedure, rather suggest a full lymphadenectomy (JSGO) or at most ‘accept’ SLN mapping as an alternative to full lymphadenectomy in this population (NCCN, BGCS). Furthermore, the SGO guidelines currently advocate the completion of lymphadenectomy with para‐aortic assessment. However, recent new data underline and clearly demonstrate the safety of SLN mapping for high‐risk endometrial cancer.^15^, ^16^ These studies might affect clinical practice beyond existing guidelines.

There is a consensus that when lymph node assessment is indicated and SLN mapping had failed, a full lymphadenectomy should be performed, acknowledging that lymph node status is pivotal for proper staging and for optimizing treatment outcomes for endometrial cancer patients.

The SLN mapping method is briefly described in most guidelines examined, favoring the ICG method. The SGO guidelines also provide a review on different methods available and studies performed, with elaboration on the products and dosages practiced. This probably unanimous ICG preference is important for standardizing the procedure of SLN mapping in different centers and, therefore results of further studies could be better generalized. Similarly, the cervix is the preferred site of injection according to all guidelines examined.

The guidelines advocate for ultrastaging, yet no uniform protocol is advised. This lack of standardization might hamper the generalizability of study results. Nevertheless, not restricting a specific protocol is in line with the varied available resources across different centers.

In cases of positive SLNs, para‐aortic lymph node sampling was suggested in most guidelines, at the discretion of the attending surgeon. Some guidelines offer imaging as an alternative to surgery in evaluating para‐aortic node involvement.

Our comparison of national guidelines regarding SLN mapping for endometrial cancer could help practitioners synthesize the data available in different countries of practice and might aid practitioners in countries where no formal guidelines are available. The inspection of national guidelines allows the practitioner to adopt recommendations from one set of guidelines that might be missing in another. Moreover, areas of controversy among guidelines are important to underline for future research and perhaps reappraisal. In countries where guidelines are not available or are based on scarce references, providing the major professional organization's spectra may be of value.

This study had some limitations. We cannot account for the methodology of how each organization reached their recommendations or decided which references on the issue to include, what will constitute a recommendation, and it was not always clear whether one organization was aware of the other organizations' recommendations.

In summary, international guidelines on SLN mapping are comparable, with major points of interest being a consensus. Nevertheless, there are noticeable variations in some information provided, references cited, and recommendations made. Clinicians may rely on issues of consensus among the different guidelines, whereas choosing a local policy in areas of controversy should take place with a proper discussion and acknowledgments of different guidelines' recommendations.

## AUTHOR CONTRIBUTIONS

All authors have accepted responsibility for the entire content of this submitted manuscript and approved submission. AD, GL, and TP designed the study, wrote the manuscript, and revised the manuscript prior to submission. LK, BB, and RM curated the data, performed the analysis, and edited the revisions.

## CONFLICT OF INTEREST

The authors have no conflicts of interest.

## Data Availability

Research data are not shared.
